# Telocytes in the Normal and Pathological Peripheral Nervous System

**DOI:** 10.3390/ijms21124320

**Published:** 2020-06-17

**Authors:** Lucio Díaz-Flores, Ricardo Gutiérrez, Mª Pino García, Sara Gayoso, Emma Gutiérrez, Lucio Díaz-Flores, José Luis Carrasco

**Affiliations:** 1 Department of Basic Medical Sciences, Faculty of Medicine, University of La Laguna, 38071 Tenerife, Spain; histologia54@gmail.com (R.G.); pilargon59@gmail.com (S.G.); emgutierrezgonzalez@gmail.com (E.G.); ldfvmri@yahoo.com (L.D.-F.J.); jcarraju@gmail.com (J.L.C.); 2 Department of Pathology, Eurofins^®^ Megalab–Hospiten Hospitals, 38100 Tenerife, Spain; mpgarcias@megalab.es

**Keywords:** telocytes, nerves, Meissner corpuscles, appendicular neurogenic hyperplasia, gallbladder neurogenic hyperplasia, peripheral nervous system tumours

## Abstract

We studied telocytes/CD34+ stromal cells in the normal and pathological peripheral nervous system (PNS), for which we reviewed the literature and contributed our observations under light and electron microscopy in this field. We consider the following aspects: (A) general characteristics of telocytes and the terminology used for these cells (e.g., endoneurial stromal cells) in PNS; (B) the presence, characteristics and arrangement of telocytes in the normal PNS, including (i) nerve epi-perineurium and endoneurium (e.g., telopodes extending into the endoneurial space); (ii) sensory nerve endings (e.g., Meissner and Pacinian corpuscles, and neuromuscular spindles); (iii) ganglia; and (iv) the intestinal autonomic nervous system; (C) the telocytes in the pathologic PNS, encompassing (i) hyperplastic neurogenic processes (neurogenic hyperplasia of the appendix and gallbladder), highly demonstrative of telocyte characteristics and relations, (ii) PNS tumours, such as neurofibroma, schwannoma, granular cell tumour and nerve sheath myxoma, and interstitial cell of Cajal-related gastrointestinal stromal tumour (GIST), (iii) tumour-invaded nerves and (iv) traumatic, metabolic, degenerative or genetic neuropathies, in which there are fewer studies on telocytes, e.g., neuroinflammation and nerves in undescended testicles (cryptorchidism), Klinefelter syndrome, crush injury, mucopolysaccharidosis II (Hunter’s syndrome) and Charcot–Marie–Tooth disease.

## 1. General Characteristics of Telocytes and Terminological Introduction

Telocytes (TCs), located in the interstitium of many tissues, were described by Popescu and Faussone-Pellegrini in 2010 [1]. These authors identified a stromal cell type, which shows a triangular or ovoid somatic body and several (two to five) long, slender, moniliform cytoplasmic processes (telopodes) with thin segments (podomeres) and dilated portions (podoms) [1,2,3]. TCs are a heterogenous population [4,5] and express CD34 and PDGFRa, among other markers. Several roles have been hypothesized for TCs in tissue homeostasis, morphogenesis, regeneration and repair, including intercellular communication with the integration of tissue components by cell-to-cell-signalling or extracellular shedding vesicles and paracrine molecules [6,7,8,9,10,11,12,13,14,15,16], the control and organisation of the extracellular matrix [15], the creation of microenvironments within the tissues [17,18,19], structural support [17,20,21,22,23,24,25], endocytosis with the internalization of small particles [9], the control and regulation of other cell types [26], guidance to cell migration during development and the contribution of scaffolds [14,17,27,28,29,30,31], immunomodulation and immunosurveillance [27], the inhibition of apoptosis (inhibition of oxidative stress and prevention of cellular ageing) [26,32], neurotransmission (e.g., contribution of slow waves generated by interstitial cells of Cajal) [10,15,25,33,34,35] and the modulation of stem cells (control of their growth and differentiation) [21,27,36,37,38,39,40,41,42,43]. In addition, TCs have mesenchymal stromal cell properties, which play an important role during repair and tumour stroma formation [18,19,37,38,39,44].

In the peripheral nervous system, TCs have been termed endoneurial stromal cells, endoneurial fibroblasts, endoneurial fibroblast-like cells, capsular fibroblasts, CD34+ endoneurial cells, dendritic endoneurial cells, endoneurial mesenchymal cells, nerve mesenchymal precursor-like cells and so forth. However, among other procedures, ultrastructural studies have clearly demonstrated that TCs are different from fibroblasts [1,2,3]. Likewise, it is widely accepted that cells ultrastructurally identifiable as TCs largely correspond to the CD34+ stromal cells (TCs/CD34+SCs) observed in light microscopy [43].

## 2. TCs in the Normal Peripheral Nervous System

In this section, we considered the presence and characteristics of TCs (TCs/CD34+SCs) in nerves, nerve fibres, free nerve endings, some sensory receptors, with and without specific structures, ganglia and the autonomic nervous system in the digestive tract.

### 2.1. TCs (TCs/CD34+SCs) in Nerves

**Figure 1:**
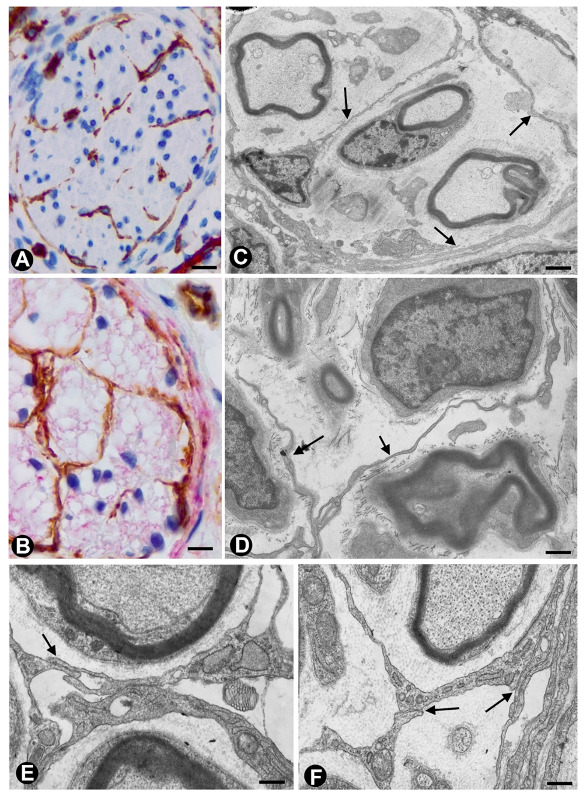
In peripheral nerves, telocytes (TCs)/CD34+SCs are observed in the epi-perineurium and endoneurium under light (**A,B**) and electron microscopy (**C**–**F**). It should be noted that in B, the TCs (brown) are arranged, underlying the perineurial cell layer (perineurial cells: red) in the epi-perineurium. C–F: Ultrastructural characteristics of endoneurial TCs, in which long, thin telopodes (C and D, arrows) and homocellular junctions (E and F, arrows) are seen. A and B: sections immunostained with anti-CD34 (A), and double-immunostained with anti-CD34 (brown) and epithelial membrane antigen (EMA) (red) (haematoxylin-stained nuclei). C–F: Ultrathin sections. Uranyl acetate and lead citrate. Bar: A, 60 µm; B, 40 µm; C, 2 µm; D, E, F, 1 µm.

In nerves, TCs (TCs/CD34+SCs) are located in the endoneurium (endoneurial cells, endoneurial fibroblasts, endoneurial dendritic cells, endoneurial mesenchymal cells) (Figure 1), although they are also present in the epi-perineurium (Figure 1A,B), together with other cell types (e.g., perineurial cells) (Figure 1B). In immunostaining with anti-CD34 in light microscopy and ultrastructurally, endoneurial TCs show long, interdigitating, moniliform telopodes (Figure 1E,F) and homocellular (Figure 1E,F) and heterocellular junctions. The telopodes extend into the endoneurial space around Schwann cells (Figure 1C–F). In the epi-perineurium, TCs are arranged on both sides of the layers formed by the perineurial cells and can occasionally be intermixed with these cells. In addition to CD34 positivity, TCs express PDGFRa [14] and PDGFRb [45] in nerves. Described in the peripheral nerve, nerve sheath tumours and related lesions, these cells have been considered immunophenotypically distinct from fibroblasts and Schwann cells [46,47,48], assimilable to TCs [49], with the capacity for collagen synthesis, phagocytosis (including myelin degradation), inflammatory response and immune surveillance [45,50,51], and originating from the neural crest [45,52]. These CD34 and PDGFRa-positive cells increased in number (about three-fold) after nerve injury [44]. TCs can also be observed in small nerves and isolated nerve fibres.

### 2.2. TCs (TCs/CD34+SCs) in Sensory Nerve Endings

TCs (TCs/CD34+SCs) can be observed in some free nerve endings and in non-encapsulated and encapsulated sensory corpuscles.

The spatial relationship of TCs with nerve endings has been described in numerous locations [2,30,35,53,54,55,56,57,58,59,60,61]. Thus, their telopodes are located in close proximity to the nerve endings. 

**Figure 2:**
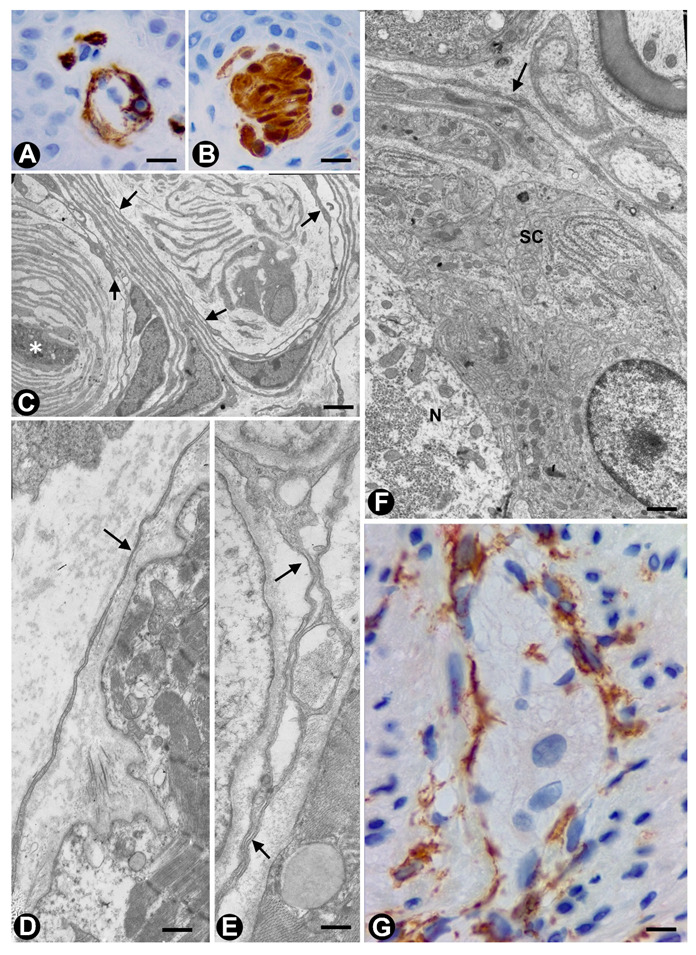
Examples of TCs in some sensory receptors, ganglia and the autonomic nervous system in the digestive tract (**A**–**C**). In Meissner corpuscles, TCs are observed in the capsule (A and C), showing CD34 expression (A, brown) and the characteristic ultrastructure (C, arrows). Schwann cells, with S100 expression (B, brown), are observed. It should be noted that in C, a nerve fibre (asterisk) surrounded by Schwann cells can be observed. (**D**,**E**) In neuromuscular spindles, TC telopodes (arrows) are shown around striated muscle cells. (**F**) A TC telopode (arrow) between a neuronal–glial unit (Neuron: N and satellite glial cell: SC) and nerve fibres in a spinal ganglion. (**G**) TCs expressing CD34 (brown) in an appendiceal myenteric ganglion. A, G: Sections stained with anti-CD34. B: Section stained with anti-S100. C–F. Ultrathin sections. Uranyl acetate and lead citrate. Bar: A, B, 60 µm; C, 2 µm; D, E, F, 1 µm; G, 40 µm

In Meissner corpuscles, TCs (TCs/CD34+SCs) form a complete or incomplete capsule (Figure 2A). Glut-1+perineurial cells do not participate in this capsule [62]. The intensity of immunoreactivity for TCs/CD34+SCs can decrease or disappear with ageing [62]. In the corpuscle, S100 positive, flattened support cells arranged like stacks of coins are seen (Figure 2B), and under electron microscopy, the TC telopodes are observed around groups of these cells (Figure 2B).

In Pacinian corpuscles, TCs (TCs/CD34+SCs) are arranged in a thin layer around the Schwann cells that surrounds the central axon. For García-Piqueras et al., 2017 [63], this layer has functional relevance since it divides the Pacinian corpuscle into two distinct compartments: inner or neural (Schwann cells and axon) and outer or non-neural (perineurial cells).

In neuromuscular spindles, TCs (TCs/CD34+SCs) are observed in the internal and external capsules [17]. In the internal capsule, the telopodes are located around intrafusal, striated muscle cells (Figure 2D,E), nerve fibres and vessels. In the external capsule, TCs (TCs/CD34+SCs) form their innermost and (partially) outermost layers. The provision of a mechanical support and the formation of a special microenvironment (glycosaminoglycans in the subcapsular and intrafusal spaces), which could facilitate the control of muscle tone and motor activity, have been suggested among other functions [17].

### 2.3. TCs in Ganglia 

TCs have been described in the human trigeminal ganglion in close vicinity to microvessels and nerve fibres around the neural–glial units [64,65]. We also observed telopodes of TCs arranged between satellite glial cells (amphicytes) and nerve fibres in the periphery of the spinal ganglion (Figure 2F) (non-published observation).

### 2.4. TCs (TCs/CD34+SCs) in the Autonomic Nervous System of the Digestive Tract

The digestive tract (above the enteric wall) is an ideal anatomic region to understand TC characteristics, arrangement and functions [17,18,19,20,25,33,66,67,68,69,70,71,72]. This adequacy for the study of TCs (specifically in the peripheral nervous system) is further increased in the neurogenic hyperplasias of the appendix and gallbladder, and the related processes. For this reason, images of the characteristics and relationship of TCs (TCs/CD34+SCs) can be found in the corresponding sections (see Section 3.1.1 and Section 3.1.2). In the digestive nervous system, TCs are seen in the nerves, nerve fascicles, isolated nerve fibres and ganglia (submucosal and myenteric plexus). TCs and their telopodes are therefore observed around the nerves and groups of fibres within them (compartmentalizing the nerve). Likewise, telopodes form networks that encompass small groups or isolated nerve fibres and that run parallel to the Schwann cells [71]. The ganglia are encompassed by a continuous or discontinuous layer of TCs (TCs/CD34+SCs), which can extend their telopodes within the ganglion (Figure 2G). In some of these locations, TCs and interstitial cells of Cajal appear intermingled (close spatial relationship). This finding is very evident in ganglia, although the presence of interstitial cells of Cajal in the submucosal ganglia is debated [4,67,69,73].

## 3. TCs (TCs/CD34+SCs) in the Pathologic Peripheral Nervous System (PNS)

TCs (TCs/CD34+SCs) participate in most pathological processes of the peripheral nervous system since they are an important part of its sheaths and interrelate with the other cellular components. TC (TCs/CD34+SCs) participation is generally reactive and can be extremely intense. They may therefore become one of the predominant cells in the lesion, as occurs in hyperplastic neurogenic processes and in some tumours of the peripheral nervous system. First of all, we will consider some morphologic variants of the hyperplastic neurogenic lesions, which show their reactive behaviour, as well as their homo and heterocellular relationships (e.g., with the vascular system). Then, we will examine tumours of the peripheral nervous system, in which TCs/CD34+SCs have an important role, and in tumour-invaded nerves. Finally, we contribute some examples of neuropathies in which TCs/CD34+SCs participate.

### 3.1. TCs (TCs/CD34+SCs) in Hyperplastic Neurogenic Processes

In this section, we consider two examples of processes with neurogenic hyperplasia: appendiceal neuropathies and neural proliferation in the gallbladder. The first is highly demonstrative of the local hyperplasia of the autonomic nervous system and the second of the changes in nerves, which are increased in number and size (increased nerve area), and neurogenic hyperplasia in the different layers of the gallbladder. 

#### 3.1.1. TCs/CD34+SCs in Hyperplastic Neurogenic Processes of the Appendix

In appendiceal nerve lesions, including neurogenic appendicopathy (neuroma, neurogenous hyperplasia, nerve hyperplasia), neurofibromatosis, ganglioneuroma and gangliocytic paraganglioma [74,75,76,77,78,79,80,81], we observed that TCs/CD34+SCs have a common response and that their extension and arrangement depend on the zones affected by the lesion, mucosa, submucosa or all appendiceal layers, allowing them to establish relations with different tissues.

Hyperplasia of most components of the autonomic nervous system is observed in these appendiceal neuropathies, including nerve fibres (individually and forming varying sized fascicles), Schwann cells, neurons and TCs. Vessels, adipocytes, smooth muscle cells and inflammatory/immunitary cells associated with these components. Generally, TCs/CD34+SCs are observed in high numbers and with numerous processes, resulting in a demonstrative morphology. They show a triangular or spindle body with a small somatic cytoplasm, which may be increased, and slender moniliform telopodes. TCs/CD34+SCs form labyrinthine systems—sometimes very intricate—in which they connect with each other or with other tissue components. Thus, TC/CD34+SC telopodes extend and surround independent nerve fibres (around Schwann cells) or small groups of them, isolated neurons between the nerve fibres or forming groups, smooth muscle cells of the muscularis mucosae and lamina propria, adipocytes, varying sized vessels and macrophages or other inflammatory/immune cells. Frequently, telopodes of the same cell are observed extending to different structures (e.g., vessels and nerve fibres). The increased appendiceal nerves also present numerous TCs/CD34+SCs arranged in the epineural layer and within the nerve, delimiting nerve fascicles or nerve fibres.

**Figure 3:**
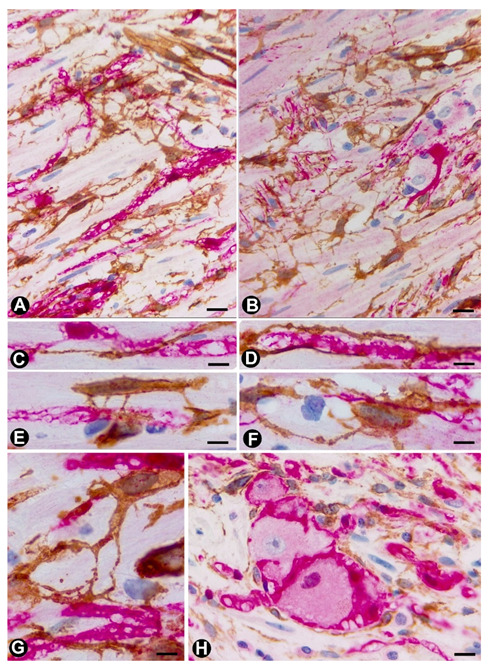
TCs/CD34+SCs (brown) around appendiceal hyperplastic nerve fibres (Schwann cells and axons) (red) (**A**–**G**) and neuronal–glial units (**B**,**H**). Sections stained with anti-CD34 (brown) and anti-S-100 (red) (A, C–E and G and H) and anti-CD34 and anti-neurofilaments (B and D). Numerous fusiform or stellate TCs and their telopodes (brown) are observed around the aforementioned structures. The TCs and telopodes follow the path of the nerve fibres and establish contact with TCs of other nerve fibres and smooth muscle cells. It should be noted how thinner telopodes can originate from the somatic region of the TCs (Figure 3E) or from thicker and initial telopodes (Figure 3G). Bar: A, B, 20 µm; C-H, 10 µm

**Figure 4:**
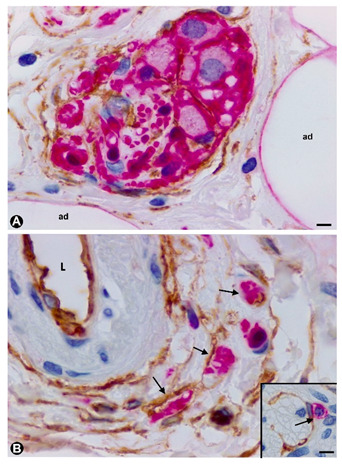
TCs/CD34+SCs around nerve fibres (Schwann cells and axons) and neuronal–glial units growing in the appendiceal adipose and connective tissues, and in the adventitia of blood vessels in the presence of mast cells. Sections double-immunostained with anti-CD34 (brown) and anti-S100 (red) (**A**), anti-CD34 (brown) and anti-neurofilaments (red) (**B**) and anti-CD34 (brown) and c-kit (red) (insert in B). A: TCs/CD34+SCs (brown) are observed around neuronal–glial units between adipocytes (ad). It should be noted that the satellite glial cells are stained in red. B: Nerve fibres between the TCs/CD34+SCs of blood vessel adventitia (L: vessel lumen). Insert of B: A c-kit immunostained mast cell (red) associated with a TC/CD34+SC) (brown). Bar: A, 20 µm; B, 40 µm; insert of B, 10 µm.

The aforementioned findings are well demonstrated in double-immunostaining (anti-CD34 and anti-S100; anti-CD34 and anti-neurofilaments) (Figure 3 and Figure 4) (non-published observations). Thus, long, thin telopodes of numerous fusiform or stellate CD34-positive TCs are seen around S100-positive Schwann cells in myriad nerve fibres and their accompanying neurons (neural–glial units), extending between smooth muscle cells of the appendiceal lamina propria (Figure 3A–H). TCs/CD34+SCs are also observed around S100-positive Schwann cells in the nerve fibres and neuronal–glial units growing between the connective and adipose tissues in the submucosa and serosa (Figure 4A), or between the vessel adventitia (Figure 4B). Likewise, the multiple relations that each TC can establish are also demonstrated with these procedures (Figure 3 and Figure 4). The characteristics of telopodes vary: they can be thicker in an initial zone, continuing in one or more filiform processes (Figure 3G), or filiform because they leave the somatic region of the cell (Figure 3E). In addition, c-kit-positive mast cells, frequently associated with TCs, are also observed (Figure 4B, insert). These mast cells must be distinguished by c-kit-positive interstitial cells of Cajal, which we did not observe in this type of process.

#### 3.1.2. TCs/CD34+SCs in Neurogenic Hyperplasia of Gallbladder

**Figure 5:**
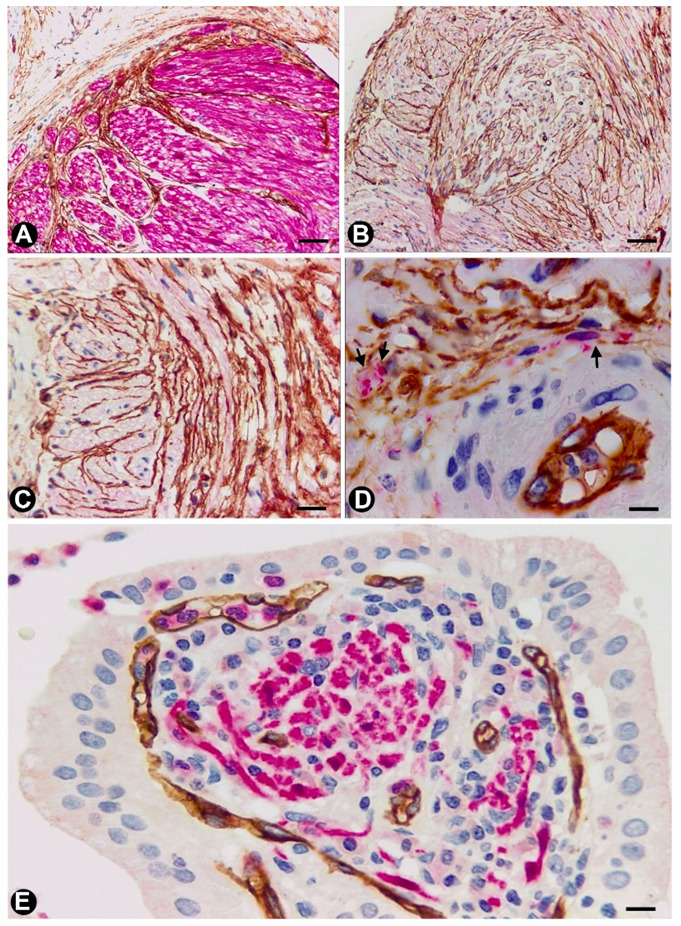
TCs in the neurogenic hyperplasia of gallbladder. (**A**) Presence of TCs/CD34+SCs (brown) around and within a thick nerve in the gallbladder wall. Schwann cells expressing S-100 (red) are seen. (**B**,**C**) TCs/CD34+SCs (brown) between fascicles and independent nerve fibres arranged in different directions within the nerves. (**D**) Nerve fibres (expressing neurofilaments, red) between TCs/CD34+SCs (brown) in the blood vessel adventitia. (**E**) Nerve fibres in the chorion of the mucosa. The absence of TCs/CD34+SCs is noted. A and E: Sections double-immunostained with anti-CD34 (brown) and anti-S100 (red). B and C: Sections immunostained with anti-CD34 (brown). D: Section double-immunostained with anti-CD34 (brown) and anti-neurofilaments (red). Bar: A, C, 80 µm; B, 100 µm; D, 20 µm; F, 30 µm.

In some cases of uncomplicated symptomatic gallstone disease, nerves can increase in number and size [82]. Likewise, the presence of numerous nerve trunks in the expanded subserosal layer has been described in adenomyomatous hyperplasia of the gallbladder with perineural invasion and in gallbladders with multiple venous and arterial thrombosis [83,84,85]. We have observed cases with a marked presence of different sized nerve trunks (some very thick) in the wall of the gallbladder (Figure 5A–C). In these cases, numerous hyperplastic nerve fibres, grouped in fascicles or independently, extended into the chorion of the mucosa, smooth muscle of the lamina propria, perimuscular subserosal layer, serosa and adventitia of different sized vessels (Figure 5). Due to their characteristics, we considered the entity a neurogenic hyperplasia of the gallbladder (non-published observation) with numerous TCs expressing CD34. Indeed, using anti-CD34 immunostaining or double-immunostaining (CD34 and S110 or anti-neurofilaments), numerous prominent TCs/CD34+SCs were observed around (a) the layers of perineurial cells (Figure 5A,B), (b) the nerve fascicles (Figure 5A–C), which were frequently arranged in several directions within the nerve (Figure 5B,C), (c) the independent nerve fibres in the nerves (Figure 5C), and the nerve fascicles and independent fibres in the connective tissue and adventitia of vessels (Figure 5D). The nerve fibres growing into the mucosa were not surrounded by CD34+ cells (Figure 5E). An important finding was the presence or absence of TCs/CD34+SCs around the Schwann cells that covered these nerve fibres. Thus, this fact may answer the question of whether TCs/CD34+SCs, together with axons and Schwann cells, originate and extend from the nerve or are incorporated from the invaded tissue. Under normal conditions, TCs/CD34+SCs are present in the adventitia of the vessels and connective tissue (where TCs/CD34+SCs surround the newly formed fibres), but are absent in the lamina propria of the mucosa (where the newly formed fibres are not surrounded by TCs/CD34+SCs). Therefore, the findings support a local origin of TCs/CD34+SCs around the newly formed fibres.

### 3.2. TCs/CD34+SCs in Tumours and of the Peripheral Nervous System

#### 3.2.1. TCs/CD34+SCs in Neurofibromas

**Figure 6:**
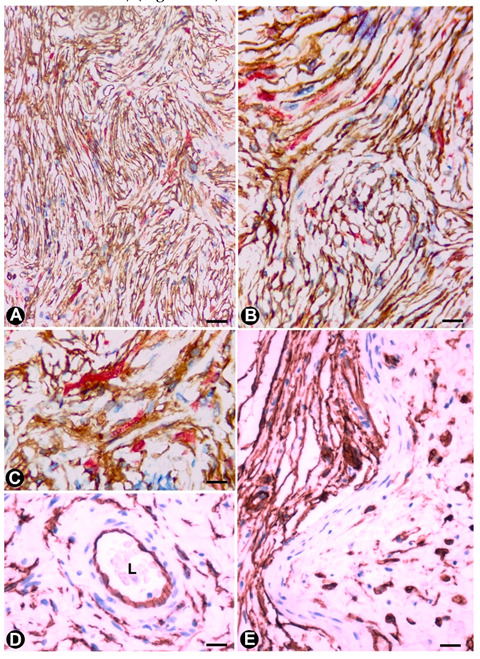
TCs/CD34+SCs in neurofibromas. (**A**–**C**) Using double-immunostaining (anti-CD34 and anti-S100), numerous TCs/CD34+SCs (brown), intermixed with Schwann cells (red) are observed forming bundles with parallel, arciform or irregular arrangement. (**D**) TCs/CD34+SCs are also seen in the adventitia of blood vessels in the tumour. (**E**) CD34+ cells (ameboid dendritic cells) in a myxoid area of a plexiform neurofibroma. D and E: Immunostained with anti-CD34. Bar: A, 80 µm; B, D, 60 µm; C, 20 µm; E, 40 µm.

An important population of TCs/CD34+SCs is present in neurofibromas, together with the remaining cellular components of the nerve (Schwann, perineurial and vascular cells) and mast cells. In all the variants of neurofibroma, including localized, diffuse and plexiform types, double staining shows that the number of TCs/CD34+SCs can be higher than the number of Schwann cells. In neurofibromas (Figure 6), TCs/CD34+SCs and Schwann cells form bundles with a parallel, arciform or irregular arrangement (Figure 6A–C), show a fusiform or stellate morphology and are associated with collagen fibres in a variable myxoid background. The adventitia of the intratumoural vessels is also formed by TCs/CD34+SCs (relation of the vessels with the principal cellular component in the tumour) (Figure 6D). 

**Figure 7:**
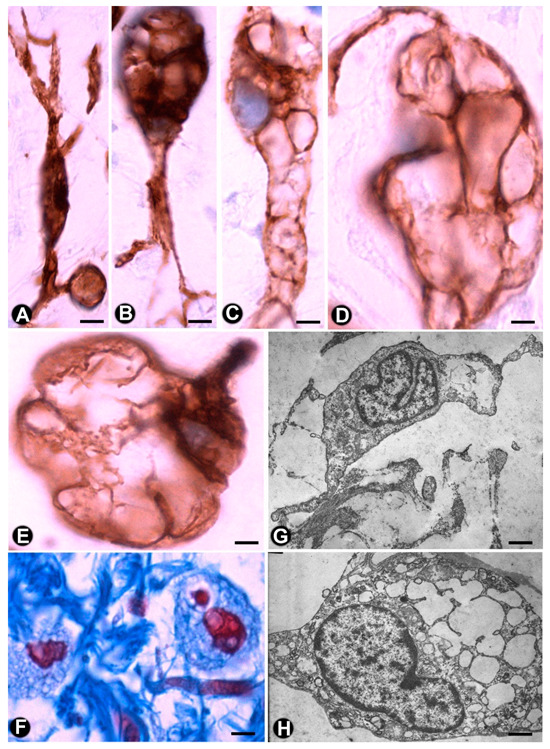
Multi-vacuolated cells (ameboid dendritic cells) in myxoid areas of plexiform neurofibromas. (**A**–**E**) CD34 expression in the vacuolated cells, which partially retain their primitive fusiform or stellate morphology (A–C) or acquire a globe-like aspect, sometimes with a piriform appearance (D and E). (**F**) Alcian blue positivity in the extracellular matrix and in the vacuolated cells (**G**,**H**) Ultrastructural characteristics of the cells in the myxoid areas. Note the intracytoplasmic vacuoles and how one cell retains some processes (G), while the other acquires a globoid-like aspect (H). A–E: Anti-CD34 immunostaining. F: Alcian blue staining. G and H: Ultrathin sections. Uranyl acetate and lead citrate. Bar: A–E, 5 µm; F, 10 µm; G, H, 0.5 µm.

In the plexiform type of neurofibroma, the myxoid deposits can be prominent (Figure 6E), and TCs/CD34+SCs increase their somatic size, showing multiple intracytoplasmic vacuoles (Figure 7a–e). In these conditions, TCs/CD34+SCs conserve their elongated aspect (Figure 7A–C) or frequently acquire an oval or round morphology, though they can still present some processes (piriform or irregular aspect) (Figure 7D,E). The intracytoplasmic vacuoles and the extracellular matrix present positivity for Alcian blue (Figure 7F). In the electron microscopy, TCs/CD34+SCs show an indented nucleus and a vacuolated cytoplasm (Figure 7G,H).

For the CD34+ cells in neurofibromas and in Antoni B zones of neurilemoma, some authors use the term ‘ameboid dendritic CD34+ cells’ [47]. CD34 expression has also been demonstrated in the multinucleated floret-like cells sporadically seen in neurofibromas, which suggests a reactive change in the endoneurial cells [86]. Cells expressing CD34 or S100 are reduced or absent in malignant peripheral nerve sheath tumours (MPNSTs) [87].

#### 3.2.2. TCs/CD34+SCs in Schwannomas

**Figure 8:**
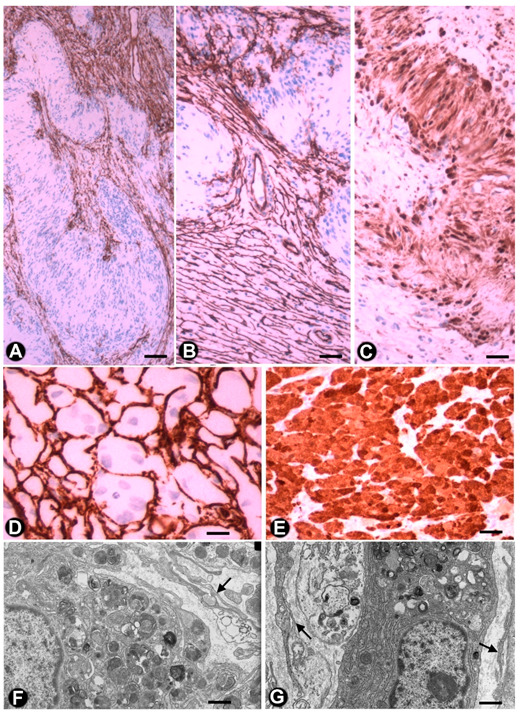
TCs/CD34+SCs in schwannomas and granular cell tumours. (**A**–**C**) A schwannoma in which numerous TCs/CD34+SCs (brown) (A and B) are arranged around groups of Schwann cells, which form Verocay bodies (A and B) and express S100 (brown) (C). D and E: Granular cell tumour, in which TCs/CD34+SCs) (brown) (**D**) surround granular cells (granular S100-positive Schwann cells) (brown) (**E**). (**F**,**G**) Ultrastructural characteristics of the granular cells in whose environment some telopodes are observed (arrows). A to E: Sections immunostained with anti-CD34 (brown) (A, B, D) and anti S-100 (brown) (C and E). F and G: Ultrathin sections, Uranyl Acetate and Lead citrate. Bar: A, B, C, E, 80 µm; D, 60 µm; F, G, 0.5 µm.

TCs/CD34+SCs are observed in Antoni B zones of schwannomas and have been described in this location as endoneurial fibroblasts or CD34-positive fibroblasts [46,88,89]. TCs/CD34+SCs are spindle, stellate or globoid, resembling those described in neurofibromas (see above). In one case of schwannoma, we observed (non-published observations) varying sized strands of TCs/CD34+SCs between numerous cellular groups or lobules with characteristics of the Antoni A zone (including the presence of Verocay bodies) (Figure 8A–C). The strands of TCs/CD34+SCs contained most of the tumour vascularization and their limit with Antony A zone groups was regular or irregular, with some strands of TCs/CD34+SCs penetrating the Schwann cell groups (Figure 8A,B).

#### 3.2.3. TCs/CD34+SCs in Granular Cell Tumour 

In the granular cell tumour (granular cell schwannoma, Abrikossoff tumour), which originates from Schwann cells, TCs/CD34+SCs are observed in light and electron microscopy (Figure 8D,F,G), surrounding groups of characteristic granular tumoural cells (S100+ granular Schwann cells, Figure 8E). Interestingly, in a subependymal giant cell astrocytoma with granular cells, we observed interstitial cells with ultrastructural characteristics of TCs (not shown).

#### 3.2.4. TCs/CD34+SCs in Nerve Sheath Myxoma

**Figure 9:**
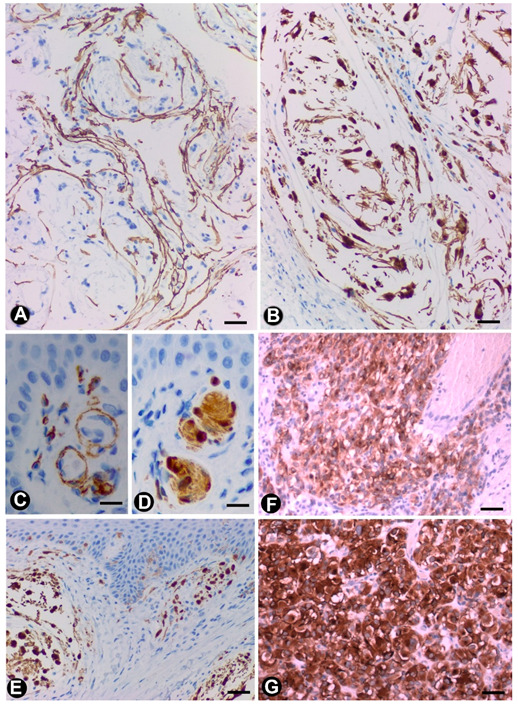
TCs/CD34+SCs and S-100+ cells in nerve myxomas (**A**–**E**), and CD34 and c-kit (CD-117) expression in gastrointestinal stromal tumours (GISTs) (**F**,**G**). A and B: Myxoid lobules in a nerve sheath myxoma, in which long, thin telopodes of spindled TCs/CD34+SCs are predominantly arranged in their periphery (A) surrounding S100**^+^** Schwann cells (B). (C–E) Increased number of Meissner corpuscles in the papillary dermis above the tumour (C, expressing CD34, and D, expressing S-100) and lobules of the lesion near the epidermis, showing a morphology reminiscent of Meissner corpuscles. (E). F and G: Expression of c-kit (F) and CD34 (G) in cells of a GIST. A, C and G: Anti-CD34 immunostaining. B, D and E: Anti-S100 immunostaining. F: c-kit immunostaining. Bar: A, B, E–G, 100 µm; C, D, 60 µm.

TCs/CD34+SCs are described as an associated, reactive component in nerve sheath myxoma, considered either a myxoid/hypocellular variant of neurothekeoma [90] or a distinctive S-100-positive myxoid peripheral nerve sheath tumour [91,92]. The lesion presents multiple myxoid lobules in the dermis, and TCs/CD34+SCs are observed in low to moderate numbers within and/or around the lobules (Figure 9A) formed by S-100-positive Schwann cells (Figure 9B). Schwann cells frequently contain a peripherally displaced, oblong nucleus and two to three cytoplasmic processes, which resemble cephalopod limbs, whereas TCs/CD34+SCs acquire a spindled aspect, with very long processes (Figure 9A). In some cases, there is an increase in Meissner corpuscles in the papillary dermis next to the sheath myxoma (Figure 9C,D), and occasional groups or lobules of the lesion show a morphology that resembles Meissner corpuscles (Figure 9E), mainly in the characteristics and arrangement of the TCs/CD34+SCs (spindled aspect and peripheral arrangement, Figure 9A) and Schwann cells (principal and central component with eccentric nuclei and processes like stacks of coins or cephalopod limbs (Figure 9B). These findings suggest a possible histogenic relationship between Meissner corpuscles and sheath nerve myxomas. 

#### 3.2.5. TCs/CD34+SCs in Gastrointestinal Stromal Tumours (GISTs)

GISTs frequently express C117 (kit protein) (Figure 9F) and CD34 (Figure 9G). Indeed, in a review of 150 cases, 90% of tumours were positive for CD117 and 50% for CD34. Therefore, most authors consider that GISTs derive from, or differentiate towards, the interstitial cells of Cajal (ICC) lineage [93,94,95,96,97]. Likewise, TCs and PDGFRa, a marker of TCs [33,98,99] are related to GISTs (familial PDGFRa-mutation syndrome) [94,100,101,102].

### 3.3. TCs/CD34+SCs in Tumour-invaded Nerves

**Figure 10:**
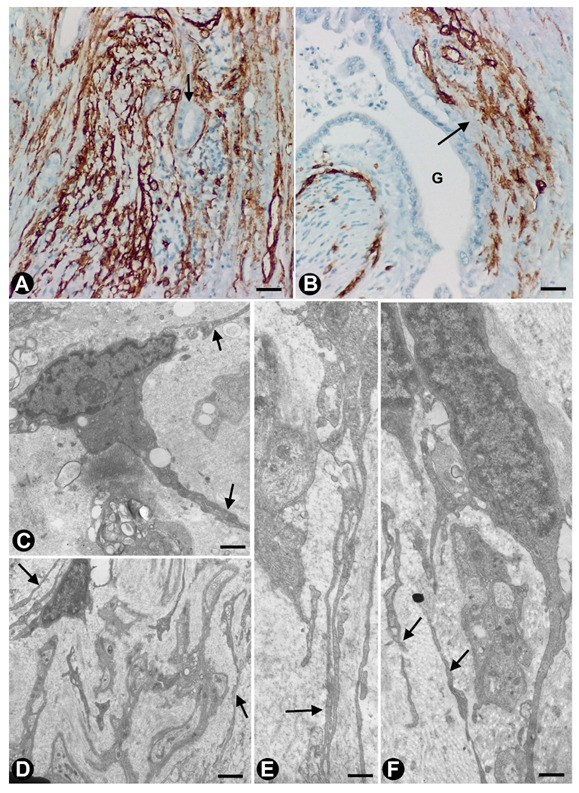
TCs/CD34+SCs in tumour-invaded and injured (compressed) nerves, and in nerves of the testicle affected by Klinefelter syndrome. (**A**) An adenocarcinoma-invaded nerve (arrow = neoplastic gland) in the gallbladder with a marked increase in the number of TCs/CD34+SCs (brown). (**B**) A nerve (arrow) next to a neoplastic gland (g) also shows hyperplastic TCs/CD34+SCs. (**C**) Telopodes of a TC (arrows) in a compressed nerve. (**D**–**F**) Ultrastructural characteristics of TCs (arrows) around nerve fibres in the testicles of a patient with Klinefelter syndrome. A and B: CD34 Immunostaining. C–F: Ultrathin sections. Uranyl acetate and lead citrate. Bar: A, B, 80 µm; C, E, F, 1 µm; D, 2 µm.

We have observed a marked reactive response of TCs/CD34+SCs in nerves with perineural invasion by adenocarcinomas (non-published observation). Thus, TCs/CD34+SCs within and around the invaded nerves appear increased in number and size (Figure 10A). The response can develop in immediate contact with the invasive neoplastic glands or distanced from them. In addition, this reactive phenomenon has also been observed in nerves close to neoplastic glands (Figure 10B), although invasion in other areas of the nerve cannot be excluded.

### 3.4. TCs/CD34+SCs in Other Pathologic Processes of the Peripheral Nervous System

In the peripheral nervous system, TCs/CD34+SCs can play a role in processes other than those outlined above, including inflammatory, traumatic, metabolic, immunologic and genetic nerve diseases. In these processes, the relationship between Schwann cells/myelin, axons, macrophages and endoneurial TCs is important, especially the membrane-bound and soluble signalling [103,104]. Below, we provide examples contributed by other authors, along with our observed but unpublished examples. Thus, TCs/CD34+SCs have been associated with neuroinflammation in the peripheral nerve [103,104] and with Charcot–Marie–Tooth disease [105,106]. They participate in myelin degradation [51] and in the resolution or location of endoneurial oedema in the sub-perineurial space following nerve crush injury [107]. Endoneurial progenitors expressing PDGFRa can participate in heterotopic ossification [108]. Under electron microscopy, membrane-bound, clear vacuoles have been described in the peripheral nerve endoneurial cells in mucopolysaccharidosis type II (Hunter’s syndrome) [109]. The authors considered these cells as endoneurial fibroblasts, which incompletely encircled the Schwann cells and amorphous material. In our opinion, the affected endoneurial cells in this work meet the ultrastructural characteristics of TCs.

In compressed nerves, we observed TCs with lipid droplets around modified nerve fibres (Figure 10C). However, myelin engulfment was only evidenced in macrophages and Schwann cells. In undescended testicles (cryptorchidism) and in the testicles of patients with Klinefelter syndrome, in which nerve fibres can be observed in the tubule walls [110,111], we identified TCs with their telopodes extending around increased and modified nerve fibres (Figure 10D–F).

## 4. Conclusions

We have reviewed the current state of knowledge about the presence, characteristics and arrangement of TCs/CD34+stromal cells in the normal and pathological peripheral nervous system (PNS), including light and electron microscopic studies in nerves, sensory nerve endings, ganglia and the intestinal autonomic nervous system, as well as in PNS tumoural and non-tumoural diseases. Further studies are required on TCs in other pathological processes of PNS, mainly in inflammatory, immunologic and genetic nerve diseases, in which intercellular communication between TCs, Schwann cells, macrophages, mast cells and lymphocytes can have an important role.
